# A practical guide to sample preparation for immunoprecipitation coupled with mass spectrometry analysis of *C. elegans* proteins

**DOI:** 10.17912/micropub.biology.002087

**Published:** 2026-04-24

**Authors:** Jian-Hua Wang, Meng-Qiu Dong

**Affiliations:** 1 College of Life Sciences, Capital Normal University, Beijing 100048, China; 2 National Institute of Biological Sciences (NIBS), Beijing 102206, China; 3 Tsinghua Institute of Multidisciplinary Biomedical Research, Tsinghua University, Beijing 102206, China

## Abstract

The success of i mmuno p recipitation coupled with m ass s pectrometry (IPMS) analysis is tied to the amount of sample input and the expression level of the bait protein in the sample. Here, we selected six *C. elegans* proteins— VIT-2 , LMN-1 , ALG-1 , AAK-2 , DAF-16 , and PQM-1 —to represent high, medium, or low expression levels; each protein had a GFP tag knocked in at the endogenous gene locus. We varied the amount of input sample and analyzed the effect on IPMS identification results. This allowed us to offer an optimized IPMS protocol with the input sample amount adjusted according to the expression level of a bait protein.

**Figure 1. Optimization of input material for IPMS of C. elegans proteins of different expression levels:** (A) Selection of bait proteins with graded endogenous expression levels for IPMS analysis based on spectral counts obtained from a publicly available *C. elegans* proteome dataset (Narayan et al., 2016). (B) Schematic diagram of the IPMS experimental workflow. (C‑H) Western blot analysis of six bait proteins across high, medium, and low expression levels. The amount of starting material (volume of packed worms) is indicated. Percentages in parentheses indicate the proportion of total sample loaded. “Input” refers to the whole worm lysate prior to affinity purification; “FT” (flow-through) denotes the supernatant collected after incubation of the lysate with antibody-coupled beads; “Pellet” represents the insoluble fraction obtained after centrifugation of whole worm lysate, which was analyzed to assess the extraction efficiency and solubility of proteins under the experimental conditions; “Elute” corresponds to the fraction recovered after elution of proteins from the affinity beads. (I) Spectral counts (PSM), sequence coverage (Cov), and ranking by spectral counts (Rank) of the six bait proteins identified in the IPMS dataset. Each bait protein was tested with high, medium, and low worm input amounts (in 4-fold increments), as specified in parentheses. (J-O) Protein–protein interaction networks for the six bait proteins, curated based on STRING database queries. Pre-existing STRING interactions that were also identified in our IPMS dataset are color-coded according to spectral counts (SC): blue for SC ≥ 50 and light blue for SC < 50. Gray nodes denote documented STRING interactions not detected under our experimental conditions. Newly identified interactors (i.e., those absent from the STRING database, after background protein removal, identified exclusively in individual samples across six samples with SC ≥ 100 and sequence coverage ≥ 0.5) are listed in orange. (P-U) Protein-protein interactions identified by xIPMS for the six bait proteins. For LMN-1 , the inter-molecular cross-link is mediated by the same K residues situated on two copies of the same protein.

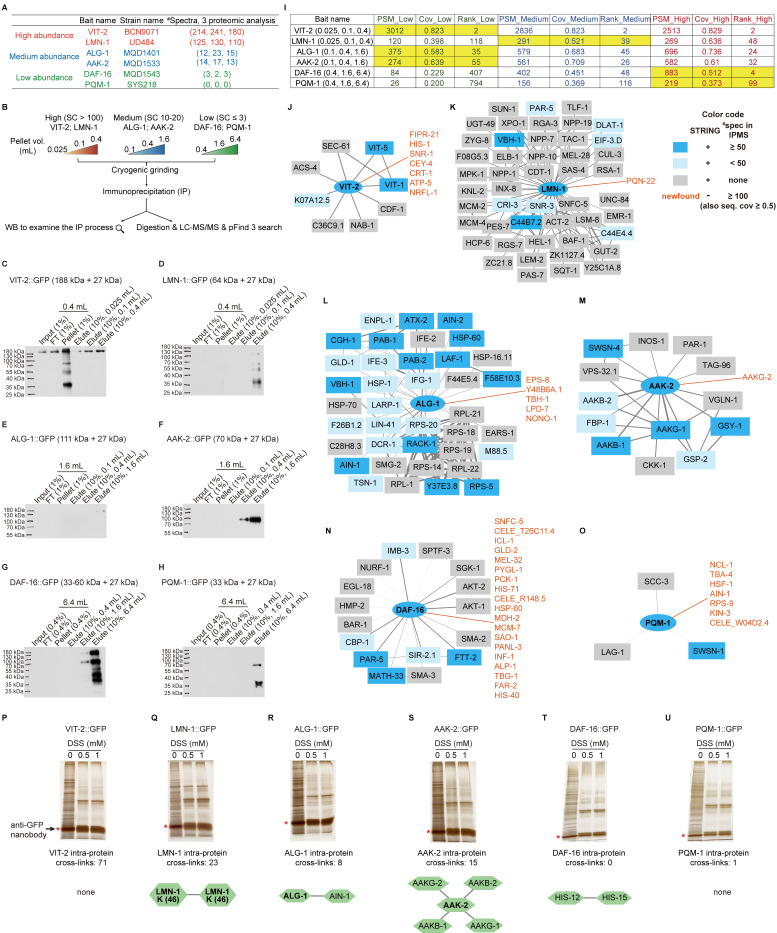



## Description

Protein-protein interactions (PPIs) underlie key cellular functions, including DNA replication, transcription, signal transduction, metabolism, and molecular transport (Greenblatt et al., 2024; Wang et al., 2022). A variety of methods are available to map PPIs in a high-throughput manner, including the ones that detect binary interactions such as yeast two-hybrid (Y2H) and the ones that analyze co‑complexes such as affinity purification-mass spectrometry (AP‑MS) (Bensimon et al., 2012; Dunham et al., 2012; Liu et al., 2026). In AP‑MS, native protein complexes are isolated through affinity capturing and then analyzed by MS (Rao et al., 2014). One commonly used AP-MS method is immunoprecipitation-mass spectrometry (IPMS), which uses antibodies to enrich protein complexes of interest, followed by MS analysis (Bonifacino et al., 2016).

A critical, yet frequently overlooked, determinant for the success of AP-MS is the amount of starting material. *C. elegans* &nbsp;proteins are often analyzed as fusion proteins with a green fluorescent protein (GFP) tag and immunoprecipitated with an anti-GFP antibody. Here, for IPMS of *C. elegans* proteins, we systematically optimized the input amount using six bait proteins representing different expression levels.

Specifically, VIT‑2, LMN‑1, ALG‑1, AAK‑2, DAF‑16, and PQM‑1 ( Figure 1A ) were selected based on their spectral counts ( *i.e.* , the number of peptide-spectrum matches, or PSM) in a publicly available&nbsp; *C. elegans* &nbsp;proteomics dataset (Narayan et al., 2016) to represent high-, medium-, or low-abundance proteins. Each protein was endogenously expressed as a GFP‑tagged fusion from its native genomic locus. Three starting amounts of worm material, differing by 4-fold increments, were tested for each bait protein (high, medium, and low abundance). The IPMS workflow is outlined in Figure 1B. Briefly, worm samples were cryogenically milled into micrometer-sized powder and lysed under conditions preserving native protein assemblies. Extracts were divided into three portions corresponding to the indicated starting volumes of packed worms. GFP-tagged complexes were immunoprecipitated using a lysine-free GFP-nanobody conjugated to magnetic beads (prepared in house). Finally, 10% of the eluate was analyzed by western blot (Ha et al., 2025), while the remainder was acetone-precipitated for MS analysis.

Western blot analysis confirmed the presence of all bait proteins, with signal intensity proportional to starting material (Figure 1C-H). Consistent with this, MS results showed that higher input amounts generally yielded higher sequence coverage of the bait protein ( Figure 1I ). For example, under high‑input conditions, VIT‑2 and DAF‑16 exhibited high spectral ranks (2 and 4, respectively). In contrast, PQM‑1 displayed a relatively low spectral rank (99) even with a large input volume (6.4 mL of worm pellet). Several factors may contribute to the low detection efficiency of certain targets: (1) substantial co‑elution of anti‑GFP nanobody—the most abundant protein in the eluate—may mask low‑abundance bait proteins and interactors; (2) suboptimal extraction efficiency—for instance, LMN-1 forms fibrous structures resistant to detergent extraction; and (3) insufficient washing during IP.

Based on MS results, we established the following criteria to determine optimal starting material: (1) bait protein spectral counts > 200; (2) bait protein sequence coverage > 0.5; and (3) no further increase in known interactors identified. An exception was made for PQM-1 , which exhibited lower sequence coverage (0.37). Applying these criteria, optimal input amounts were determined (highlighted in yellow in Figure 1I ): 0.025 mL for VIT-2 ; 0.1 mL for LMN-1 , ALG-1 , and AAK-2 ; and 6.4 mL for DAF-16 and PQM-1 . Although these thresholds are specific to the current GFP‑nanobody system, they should be a useful reference for other systems.

To benchmark our IPMS results, we compared background-subtracted interactors against known physical interactions retrieved from the STRING database (https://version-11-5.string-db.org). The reference dataset included only interactions supported by experimental evidence, classified as physical associations, with confidence scores ≥ 0.4. This analysis revealed that the number of known interactors detected did not increase linearly with input material across low-, medium-, and high-abundance bait proteins, although confidence scores of recovered interactions were generally higher under high-input conditions. The PPIs mapped under the optimized input amounts are depicted in Figure 1J –O. Interactors identified in our dataset are color-coded in blue and light blue. Gray nodes represent documented STRING interactions not detected under our experimental conditions, while newly identified interactors (i.e., those absent from STRING) are in orange letters.

While IPMS effectively identifies physical protein contacts, it cannot distinguish direct from indirect interactions or resolve structural details of contact sites. Chemical cross‑linking of proteins coupled with mass spectrometry (CXMS) addresses these limitations by providing distance restraints between cross‑linked residues, enabling conformational analysis and interface characterization (Tan et al., 2016; Wang et al., 2023; Wang et al., 2022; Yang et al., 2012). The integration of CXMS with IP—termed cross‑linking immunoprecipitation‑MS (xIPMS)—has been used to locate protein-protein contact sites in immunoprecipitated complexes (Makowski et al., 2016; Shi et al., 2015). In this study, we applied stringent filtering criteria (see Methods) and readily identified intra-molecular cross-links for each bait protein. In contrast, only a small number of inter-protein cross-links were detected ( Figure 1P- U).

The deficiency of inter-protein cross-links likely have to do with multiple factors. First, intra-molecular chemical reactions have a spatial advantage over inter-molecular ones, so inter-protein cross-links are naturally disfavored at production. Second, high-abundance proteins (e.g., the anti‑GFP nanobody) have a masking effect on low-abundance proteins, which could include authentic binding proteins of the bait or even the bait protein itself, let alone inter-protein cross-links. In our data, approximately one‑third of the top 200 proteins (by spectral counts) were consistently detected across all six samples. These abundant, probable background proteins are likely a major problem. Additionally, we applied stringent filtering criteria—if a cross-link could be intra- or inter-protein at the same time, it is treated as an intra-protein, not inter-protein cross-link. Clearly, the xIPMS protocol needs optimization. Improvement may be achieved by reducing background proteins, increasing input material, or using a cross-linker bearing an affinity tag to enrich cross-linked peptides.

Beyond input quantity, bait protein abundance and other biochemical and procedural factors critically influence the outcome of IPMS. For example, bait protein solubility and extractability—exemplified by the detergent resistance of LMN-1 —directly govern IPMS efficiency. The diversity and stability of protein assemblies also matter. For instance, weakly associated interactors are more likely to dissociate during immunoprecipitation, and stable high-abundance complexes may mask fragile low-abundance ones. Background interference from nonspecific binding or inadequate washing can markedly diminish detection sensitivity.

In conclusion, we developed a reference protocol for IPMS of *C. elegans* proteins of various expression levels. Adjustments may be needed to accommodate unique properties of individual proteins of interest.

## Methods

**Culturing and collecting worms**

(1) Seed mixed stage worms on HG solid plates at 20 °C and allow the worms to grow until the desired stage. For basic *C. elegans* growth and maintenance, please see Stiernagle (Stiernagle, 2006) and Porta-de-la-Riva, Fontrodona, et al (Porta-de-la-Riva et al., 2012).

(2) Collect worms in a 15 ml conical centrifuge tube by washing the worm plates with M9 buffer.

(3) Pellet the worms by centrifuging at 2,000 rpm at room temperature (RT) for 1 min and discard the supernatant.

(4) Perform additional 3-5 washes with M9 buffer or until the supernatant is no longer cloudy.

(5) Perform one final wash with ddH _2_ O.

(6) Move the loose worm pellet to a 1.5 mL microcentrifuge tube and spin down at 2,000 rpm at RT for 2 min. Discard the remaining supernatant to obtain a packed worm pellet and proceed to extract preparation.

NOTE: The protocol can be paused here. Worm pellets may be flash frozen in liquid nitrogen immediately and stored at -80 °C or in liquid nitrogen. Please note that worm pellets can only be thawed once and cannot be refrozen.

&nbsp;

**Extract preparation**

(1) 1-3 mL frozen worm pellets were cryogenic grinding on Mixer mill MM 400. Frequency 30/s, time 1 min, then put the grinding jar in liquid nitrogen immediately for 20 seconds.

(2) Repeat procedure (1) six times, then check the lysing efficiency of cells under microscopy, if the efficiency is not high, repeat procedure (1) twice more.

(3) Transfer the cell lysate powder to 50 ml Eppendorf tube, keep on liquid nitrogen.

(4) Add equivalent volume of freshly prepared 2x Lysis buffer to the cell lysate powder. Mix the mixture till homogenously by shaking at 4 °C.

(5) Transfer the cell lysate to a suitable centrifuge tube, keep on ice.

(6) Centrifuge at 14,000 rpm for 30 min at 4 °C, transfer supernatant to a new centrifuge tube.

(7) Centrifuge the supernatant at 14, 000 rpm for 20 min at 4 °C, transfer supernatant to a fresh tube on ice. The supernatant is now the clarified extract. Use the extract immediately for the following experiments. Save 1-3% the clarified extract for western blot detection.

&nbsp;

**Immunoprecipitation**

(1) Wash homemade anti-GFP nanobody beads: Fetch and wash 2 mg magnetic beads with 1x lysis buffer three times, centrifuge at 1,000 rpm for 1 min at 4 °C.

(2) Add the lysate to washed beads, incubate at 4 °C for one hours.

(Note: You can adjust the incubate time according to your requirement, normally, one hour is used.)

(3) Put the samples on a magnetic stand for 10 s, discard the supernatant carefully. Save 1-3% the supernatant for western blot detection.

(4) Add 1 mL wash buffer to the beads, shaking at 4 °C for 5 min, centrifuge at 1,000 rpm for 1 min. Discard the supernatant carefully.

(5) Repeat procedure (4) for 2 times.

(6) Add 100 µL elution buffer 1 to beads, incubate at 4 °C for 10 min. Centrifuge at 1,000 rpm for 1 min at RT, and aspirate 1/10 of the sample to a new tube for western blot detection. Transfer left liquids to a collecting 1.5 mL Eppendorf tube.

(7) The rest 9/10 of the sample is precipitated by 6x volume of pre-cooled acetone at -20 °C for 30 min to overnight.

(8) Spin in a refrigerated microfuge at top speed for 30 min. Aspirate most of the supernatant and leave the last drop so as not to disturb the pellet. The air dry the pellets were used for the following in-solution digestion. NOTE: The protocol can be paused here. The samples can be stored at -20 °C.

&nbsp;

**Chemical cross-linking**

(1) Preparation of chemical cross-linkers: dissolve DSS in DMSO (make fresh before each use and keep DMSO in&nbsp;a desiccator). Adjust the stock&nbsp;concentration with DMSO&nbsp;and make sure that the amount of DMSO&nbsp;in the cross-linking reaction does not&nbsp;exceed 10% by volume. (Normal stock concentration: 50 mM)

(2) On-bead cross-linking: add the cross-linker solution to&nbsp;the washed IP samples (Immunoprecipitation procure, after step (5)) at a final concentration of 0.5-1 mM, mix well.

(3) Cross-link at room temperature for 1 h.

(4) Terminate the reaction by adding 1 M ammonium bicarbonate to a final concentration of 20 mM and incubating at room temperature for 20 min.

(5) Add 100 µL elution buffer 2 to beads, incubate at 85 °C for 10 min. Centrifuge at 3,000 rpm for 1min at RT, run 1/5 of the sample on SDS-PAGE to examine the cross-linking reaction, the SDS-PAGE can be stained by Coomassie G-250 or silver stain. NOTE: silver stain is preferred when the total loading amount of protein is lower.

(6) The rest 4/5 of the sample is precipitated by 1/3 volume of 100% TCA on ice for 30 min to overnight.

(7) Spin in a refrigerated microfuge at top speed for 30 min at 4 °C. Aspirate most of the supernatant and leave the last drop so as not to disturb the pellet.

(8) Wash with 500 µL of ice-cool acetone twice. After each wash spin for 10 min at 4 °C. The air dry the pellets were used for the following in-solution digestion. NOTE: The protocol can be paused here. The samples can be stored at -20 °C.

&nbsp;

**In-solution digestion**

(1) Dissolve ~10 µg of the freshly precipitated protein sample in 15 µL of 8 M urea, 100 mM Tris, pH 8.5.

(2) Add 0.75 µL 100 mM TCEP (to 5 mM final conc.) and incubate at room temperature for 20 min.

(3) Add 0.3 µl 500 mM iodoacetamide (to 10 mM final conc.), incubate at room temperature for 15 min in the dark.

(4) Add 45 µl of 100 mM Tris, pH 8.5 (final volume = 60 µL, final concentration of urea = 2 M).

(5) Add methylamine to 20 mM to reduce the modification of carbamylation. (i.e. 1 µL 1.2 M methylamine solution, 60x)

(6) Based on the sequences of the proteins in question to decide which proteases to use. (e.g. the most widely used enzyme trypsin, add trypsin (0.5 µg/µL) at 50:1-100:1 substrate:enzyme ratio (w/w), incubate at 37 °C for 12-16 h in the dark)

(7) Quench the digestion reaction by adding 90% formic acid to a 5% ﬁnal concentration.

(8) Spin in a tabletop microcentrifuge at ≥ 13,000 rpm for 30 min, transfer the supernatant to sample vials, and freeze at -80 °C before LC-MS/MS analysis.

&nbsp;

**LC-MS/MS analysis**

**LC method**

All protein samples were analyzed using an EASY-nLC 1000 system (Thermo Fisher Scientific, Waltham, MA) interfaced with a Q-Exactive HF mass spectrometer (Thermo Fisher Scientific). Peptides were loaded on a pre-column (75 μm ID, 4 cm long, packed with ODS-AQ 12 nm-10 μm beads) and separated on an analytical column (75 μm ID, 12 cm long, packed with Luna C18 1.9 μm 100 Å resin) with a 90 min linear gradient at a flow rate of 250 nl/min as follows: 0–5% Buffer B in 1 min, 5-30% Buffer B in 69 min, 30-100% Buffer B in 10 min, 100% Buffer B for 10 min (Buffer A = 0.1% FA, Buffer B = 100% ACN, 0.1% FA).

&nbsp;

**MS method**

We used a Thermo Scientific Q-Exactive HF mass spectrometry for the data acquisition. The parameters were as follows: spray voltage 2.3 kV; capillary temperature 275 °C; data-dependent mode; full scan resolution 60,000; MS2 scan resolution 15,000; isolation window 2.0 m/z; AGC target at 3e ^6^ for FTMS full scan and 5e ^4^ for MS2 in the cross-linked samples; AGC target at 1e ^6^ for FTMS full scan and 5e ^4^ for MS2 in the non-cross-linked samples; full MS scan range 300-2000; normalized collision energy at 27%; the top fifteen most intense precursor ions were isolated for HCD MS2 with a dynamic exclusion time of 30 s; precursors with 1 ^+^ , 2 ^+^ , more than 6 ^+^ , or unassigned charge states were excluded for the cross-linked samples; precursors with 1 ^+^ , more than 6 ^+^ , or unassigned charge states were excluded for the non-cross-linked samples. Each sample was analyzed in technical duplicates.

&nbsp;

**Data analysis**

**Protein identification**

The MS data were searched against a *C.elegans* protein database (downloaded from Uniprot or WormBase) using pFind 3 with the following parameters: instrument, HCD-FTMS; precursor mass tolerance, 20 ppm; fragment mass tolerance 20 ppm; variable modification, Carbamidomethyl[C], Deamidated[N], Deamidated[Q] and Oxidation[M]; peptide length, minimum 6 amino acids and maximum 100 amino acids; peptide mass, minimum 600 and maximum 10,000 Da; enzyme, trypsin, with up to three missed cleavage sites. The results were filtered by requiring FDR < 1% at the peptide level.

**&nbsp;**

**Identification of cross-linked peptides**

For each bait protein, proteins present in the corresponding xIPMS sample were identified using pFind3 and used to generate a protein sequence database for subsequent pLink 3 search.

The search parameters used for pLink 3 were as follows: instrument, HCD; precursor mass tolerance, 10 ppm; fragment mass tolerance 20 ppm; cross-linker DSS (cross-linking sites K and protein N-terminus, cross-link mass-shift 138.068, mono-link mass-shift 156.079); cross-linker BS ^3^ (cross-linking sites K and protein N-terminus, cross-link mass-shift 138.068, mono-link mass-shift 156.079); variable modification, Carbamidomethyl[C], Deamidated[N], Deamidated[Q], Deamidated[R] and Oxidation[M]; peptide length, minimum 6 amino acids and maximum 60 amino acids per chain; peptide mass, minimum 600 and maximum 6,000 Da per chain; enzyme, trypsin, with up to three missed cleavage sites per cross-link. The results were filtered by requiring a CSM FDR < 0.01, E-value < 0.001, spectral counts ≥ 2. Cross-links outputted by pLink 3 as both inter- and intra-molecular (due to sequence homology) are treated categorically as intra-molecular. Unless there are reasons to believe otherwise.

## Reagents

**Reagents**

NaCl (Beijing Shiji)

NaOH (Beijing Shiji)

HEPES (N-(2-hydroxyethyl)piperazine-N-(2-ethanesulfonic acid; Sigma-Aldrich, cat. no. H3375)

Glycerol (AMRESCO, cat. no. 0854-1L)

Triton X-100 (AMRESCO, cat. no. 0694-500mL)

PMSF (Thermo Fisher Scientific, cat. no. 36978)

DTT (Sigma-Aldrich, cat. no. 43816)

SDS (LABLEAD, cat. no. L5750-100G)

CaCl _2_ (Fluka analitical, cat. no. 06991)

MgSO _4_ (Sigma-Aldrich, cat. no. M7506)

Glycine (AMRESCO, cat. no. 0167-5KG)

Roche® cOmplete™ EDTA-free protease inhibitor cocktail tablets (Roche, cat. no. 04693132001)

PhosStop phosphatase inhibitor cocktail tablets (Roche, cat. no. 04906837001)

SuperSignal ^TM^ West Dura Extended Duration Substrate (Thermo Fisher Scientific, cat. no. 34075)

ProteoSilver™ Plus Silver Stain Kit (Sigma-Aldrich, cat. no. PROTSIL2-1KT)

Urea (Sigma-Aldrich, cat. no. U0631)

TCEP-HCL (tris (2-carboxyethyl) phosphine (Thermo Fisher Scientific, cat. no. 20490)

Iodoacetamide (IAA) (Sigma-Aldrich, cat. no. I1149)

Methylamine (Sigma-Aldrich, cat. no. 42,646-6)

Formic acid (FA) (J. T. Baker, cat. no. 0129)

Trichloroacetic acid solution (TCA) (Sigma-Aldrich, cat. no. T0699)

Acetone (J. T. Baker, cat. no. 9002-02)

Acetonitrile (ACN), HPLC grade (Fisher Scientiﬁc, cat. no. A998)

Water, HPLC grade (Sigma-Aldrich, cat. no. V270733)

Sequencing-grade modified trypsin (Promega, cat. no. V5117)

DMSO (Sigma-Aldrich, cat. no. 34869)

Disuccinimidyl suberate (DSS) (Thermo Fisher Scientific, cat. no. 21555)

bis(sulfosuccinimidyl)suberate (BS ^3^ ) (Thermo Fisher Scientific, cat. no. 21580)

&nbsp;

**Buffers**

M9 buffer: 22 mM KH _2_ PO _4_ , 42 mM Na _2_ HPO _4_ , 86 mM NaCl, 1 mM MgSO _4_ ; sterilize by autoclaving.

2x Lysis buffer: 50 mM HEPES-NaOH pH 7.4, 300 mM NaCl, 1% Triton-X100, 10% glycerol, 2 mM PMSF, 4x Protease inhibitors: Roche® cOmplete™ EDTA-free protease inhibitor cocktail tablets, 2x Phosphatase inhibitors: Roche® PhosSTOP™ phosphatase inhibitor cocktail tablets. Prepare it freshly and keep it on ice when using.

Wash buffer: 1x Lysis buffer; prepare it freshly and keep it on ice when using.

Elution buffer 1: 0.1 M glycine-HCl buffer, pH 2.6.

Elution buffer 2: 2% SDS, 100 mM Tris-HCl, pH 8.5.

&nbsp;

**Equipments**

Mixer mill MM 400 (Retsch)

Refrigerated bench top centrifuge (Eppendorf)

Tottering Mixter (Kylin-Bell)

Vortex-Genie® 2 (Scientific Industries)

&nbsp;

**Softwares**

pFind 3 (Chi et al., 2018; Shao et al., 2021) (https://pfind.ict.ac.cn/se/pfind/#Downloads, version 3.1.6 and 3.2.2), protein identification;

pLink 3 (Chen et al., 2019) (https://github.com/pFindStudio/pLink3/releases, version 3.0.17), cross-linked peptide pairs identification;

MSFileReader 3.0 SP2 (https://github.com/pFindStudio/pLink2/wiki/FAQ#how-to-install-msfilereader), pFind 3 and pLink 3 uses MSFileReader to access RAW files;

Cytoscape (https://github.com/cytoscape/cytoscape/releases/3.10.2/, version 3.10.2), an open source bioinformatics software platform for visualizing protein interaction networks.

## Data Availability

Description: IPMS list of six baits. Resource Type: Dataset. DOI: https://doi.org/10.22002/yey2p-6hf34 Description: xIPMS list of six baits. Resource Type: Dataset. DOI: https://doi.org/10.22002/7113p-pxd27
